# Transmissive-Detected Hyperspectral Imaging for Single-Vessel-Resolution Blood Oxygen Mapping

**DOI:** 10.34133/bmef.0211

**Published:** 2025-12-16

**Authors:** Shaojun Liu, Qing Xia, Yuwei Du, Tingting Yu, Dongyu Li, Dan Zhu

**Affiliations:** ^1^ MoE Key Laboratory for Biomedical Photonics, Wuhan National Laboratory for Optoelectronics-Advanced Biomedical Imaging Facility, Huazhong University of Science and Technology, 430074 Wuhan, Hubei, China.; ^2^ Optics Valley Laboratory, 430074 Wuhan, Hubei, China.; ^3^ School of Optical Electronic Information, Huazhong University of Science and Technology, 430074 Wuhan, Hubei, China.

## Abstract

**Objective:** This study proposed a transmissive-detected hyperspectral imaging (TD-HSI) strategy for blood oxygen mapping in order to address the limitation of reflective HSI in obtaining high-resolution blood oxygen information from deep tissues. **Impact Statement:** This innovative TD-HSI has great potential in promoting noninvasive, high-resolution in vivo blood oxygen monitoring and provides a powerful tool for the study of tissue oxygenation and microcirculation diseases. **Introduction:** Oxygen saturation (SO_2_) served as a critical indicator reflecting physiological states. However, strong scattering of tissue prevents accurate SO_2_ mapping with promising resolution, which also limited the depth of reflective HSI. **Methods:** Monte Carlo simulations were employed to theoretically evaluate the deep-tissue measurement of SO_2_ between conventional reflective-detected HSI (RD-HSI) and TD-HSI. Then, in vivo TD-HSI system was used to observe the impact of hypoxia on individual arteries and veins at various locations in mice, and monitor the SO_2_ fluctuations during subcutaneous tumor growth over a 1-week period. **Results:** The simulations showed that TD-HSI remarkably extended the depth of accurate SO_2_ detection and boasted approximately 6-fold greater precision in detecting SO_2_ variations. In vivo experiments validated the efficacy of TD-HSI, demonstrating its capability to achieve SO_2_ mapping in mice skin with single-vessel resolution, a feat not possible with RD-HSI. **Conclusion:** We conducted a comprehensive evaluation of the capability of TD-HSI strategy for deep-tissue blood oxygen imaging. Our data demonstrated that TD-HSI offered substantial improvements over conventional RD-HSI in noninvasively acquiring blood oxygen information in deep tissue.

## Introduction

Oxygen is indispensable for sustaining life activities, as proper oxygen supply and utilization are crucial in preserving the normal structure and function of tissues. Abnormal blood oxygen levels are intricately linked to the emergence and progression of numerous diseases, including stroke [1], tumor [2,3], diabetic foot ulcers [4–6], and skin wounds [7,8]. Given that oxygen saturation (SO_2_) serves as a key indicator of the oxygen level in the blood [9], monitoring SO_2_ holds immense importance in diagnosing diseases and assessing prognosis.

Since oxyhemoglobin and deoxyhemoglobin have distinguished absorption spectra [10], the noncontact optical detection strategies have become an ideal method to measure SO_2_ in vivo, and some have already been used in clinical applications, such as pulse oximetry [11–13]. However, pulse oximetry is not able to provide fine distribution of SO_2_ in tissues [14], which is important because in some diseases arterial and venous SO_2_ undergo different and even opposite changes [15]. Combining spectral analysis and optical imaging, hyperspectral imaging (HSI) technique enables SO_2_ mapping in vasculature, playing an important role in various biological studies in the last decades [16–18]. However, the strong scattering of tissue hinders HSI from extracting deep-tissue information with an acceptable resolution. Although optical coherent tomography (OCT) [19,20] and photoacoustic microscopy (PAM) [21–23] combined with spectral detection are able to achieve deep tissue oxygen saturation measurements, they still suffer from systematic complexity, hardware cost, and scanning-induced time consuming, making it not as easy as wide-field HSI to spread. Therefore, it will be of great value if the low-cost, easy to integrate HSI could conquer its limitation of imaging depth.

Conventional HSI typically relies on reflective detection, where incident photons undergo strong scattering and absorption by superficial tissues. This limitation not only prevents adequate light penetration to deeper blood vessels but also leads to an unacceptably high proportion of undesirable superficial spectral information in the detected signals [24], thereby posing challenges in accurately capturing the absorbance properties of deep vasculature.

To address this limitation in imaging depth, we proposed a transmissive-detected HSI (TD-HSI) technology, capable of achieving cutaneous SO_2_ mapping at single-vessel resolution in mice. The transmissive-detected mode could minimize the interference of surface information, thereby unveiling previously undetectable deep-tissue SO_2_ information. To theoretically evaluate the deep-tissue measurement of SO_2_ between conventional reflective-detected HSI (RD-HSI) and TD-HSI, we employed Monte Carlo simulations. These simulations show that TD-HSI remarkably extends the depth of accurate SO_2_ detection and boasts approximately 6-fold greater precision in detecting SO_2_ variations. Following this, we constructed the imaging system and conducted imaging experiments on tissue phantoms, corroborating our simulation results. Finally, we applied the technique on in vivo blood oxygen imaging, where TD-HSI proved capable of distinguishing dynamic SO_2_ changes in individual cutaneous arteries and veins, details that were completely undetectable with conventional RD-HSI. Using this method, we monitored SO_2_ fluctuations during a 1-week period of subcutaneous tumor growth. Our findings revealed that SO_2_ saturation in the tumor area initially rose and then fell, while the SO_2_ in the surrounding area continued to climb throughout the observation period.

## Results

### The comparative analysis of TD-HSI and RD-HSI in SO_2_ measurement with Monte Carlo simulation

Using Monte Carlo simulations and mathematical calculations, we determined the absorption spectra of the vascular layer at varying depths, considering SO_2_ levels of 98% and 75%, corresponding to arterial and venous blood, respectively.

**Fig. 1.**
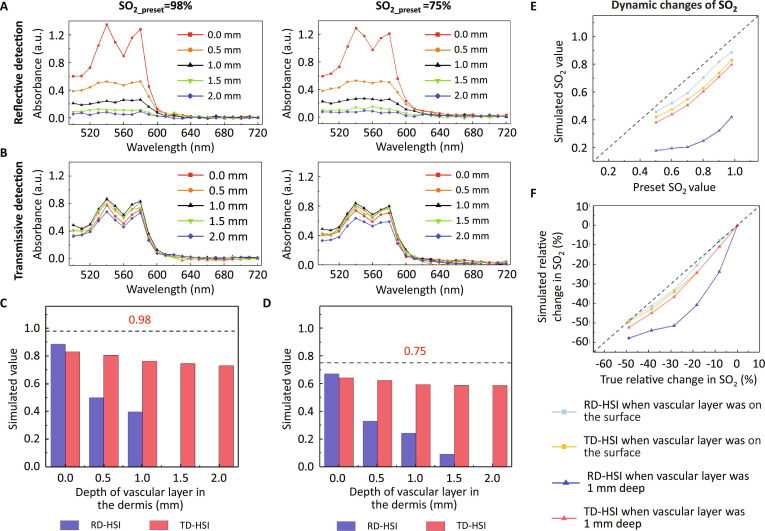
Simulation of SO_2_ and its changes measured by RD-HSI and TD-HSI. (A and B) Absorbance when vascular layer with SO_2_ of 98% or 75% was located at different depths in the tissue model under reflective detection (A) and transmissive detection (B). (C and D) Effect of vascular depth on simulated values when the SO_2_ value was preset to 98% (C) or 75% (D) for RD-HSI and TD-HSI. (E) Comparison of detection sensitivity of RD-HSI and TD-HSI when the ${\mathrm{SO}}_{2}$ value of the vascular layer at the surface or 1 mm deep gradually decreases from 98% to 50%. (F) Comparison of accuracy of RD-HSI and TD-HSI in measuring changes in SO_2_ when the SO_2_ value of the vascular layer at the surface or 1 mm deep gradually decreases from 98% to 50%.

As shown in Fig. 1A, using reflective detection mode, only when the vascular layer was located at the most superficial layer of the tissue, the detected absorption spectrum was M-shaped and consistent with the theoretical calculating value (Fig. S1), while it gradually deviated from the M-shape when the vascular layer went deeper. When the depth increased to 2 mm, it was no longer able to extract the light absorption information of the tissue. In sharp contrast, the measured absorption spectrum consistently exhibited an M-shaped pattern in transmissive detection mode, irrespective of whether the vascular layer was positioned superficially or at a depth of 2 mm (Fig. 1B).

Consequentially, with increasing vascular depth, the SO_2_ measured by RD-HSI rapidly diverged from the true value, becoming almost undetectable beyond 1-mm depth. Conversely, although TD-HSI measurement was slightly less accurate than RD-HSI measurement at superficial layer, it exhibited a relatively stable variation with depth, enabling accurate measurements even at depths up to 2 mm (Fig. 1C and D).

During the validation of SO_2_ change detection, we constructed a series of “measured value”–“preset value” curves, and the detection sensitivity could be quantified by the slope of the curves. Previous Monte Carlo simulation results (Fig. 1A) showed that, when the vessel depth exceeds 1 mm, the difference in light absorbance at different wavelengths in tissues acquired through RD-HSI notably decreased. Furthermore, the simulation results for arteries (Fig. 1C) indicated that RD-HSI is unable to effectively obtain SO_2_ value when the vessel depth exceeds 1 mm. Therefore, a maximum vessel depth of 1 mm was used to compare the detection sensitivity of RD-HSI and TD-HSI. As shown in Fig. 1E, TD-HSI and RD-HSI demonstrated comparable sensitivity at the surface. However, at a depth of 1 mm, TD-HSI clearly surpassed its counterpart in sensitivity. Notably, when SO_2_ dropped below 70%, the change became virtually undetectable. Figure 1F shows the relative changes in SO_2_ detected by RD-HSI and TD-HSI, which demonstrated similar results as above. The accuracy of RD-HSI in detecting SO_2_ change greatly decreased with the increase of the signal depth, while the accuracy of TD-HSI was 3 to 6 times higher at 1-mm depth. Therefore, our simulations clearly demonstrated that TD-HSI could detect the dynamic changes of blood oxygen level in deep tissue with higher sensitivity and accuracy compared with RD-HSI.

### In vitro SO_2_ measurement in tissue phantom

**Fig. 2.**
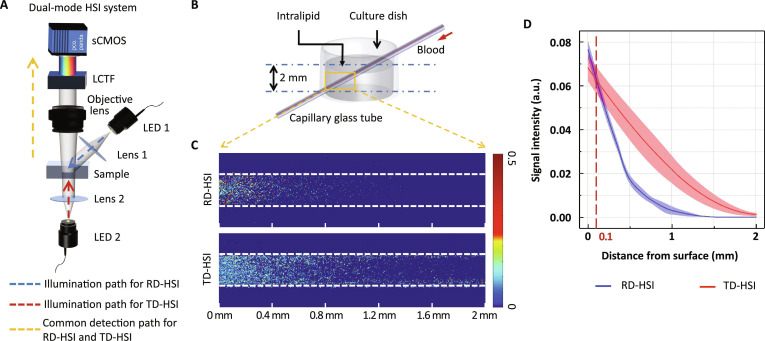
Comparison of TD-HSI and RD-HSI for blood oxygen imaging by tissue phantom experiment. (A) Schematic diagram of the dual-mode system combining conventional RD-HSI with TR-HSI microscope. (B) Schematic diagram of tissue phantom. (C) Blood oxygen distribution images of tissue phantom acquired with RD-HSI and TD-HSI. The white dashed line indicates the inner wall of the capillary glass tube. The scale line represents the distance to the surface of the intralipid. (D) Quantification of the effect of blood vessel depth on the signal intensity.

We constructed a dual-mode imaging system combining conventional RD-HSI with TD-HSI microscope and further performed tissue phantom SO_2_ imaging to evaluate their imaging capability at various depths (Fig. 2A and B). Figure 2C shows the SO_2_ mapping of the blood-filled capillary inside tissue phantom obtained with RD-HSI and TD-HSI, respectively.

As depicted in Fig. 2D, quantitative analysis revealed that RD-HSI exhibited stronger signal intensity compared to TD-HSI when vessel depth was less than 0.1 mm. However, as vessel depth increased, the signal intensity captured by RD-HSI diminished swiftly and was eventually outweighed by that of TD-HSI. Once the depth exceeded 1.5 mm, RD-HSI became unable to detect any signal, whereas TD-HSI maintained detection capability up to 2 mm. These findings underscore TD-HSI’s superiority over RD-HSI in measuring SO_2_ of deeper blood vessels, corroborating our simulation results.

### In vivo SO_2_ mapping of cutaneous vasculature in mice

**Fig. 3.**
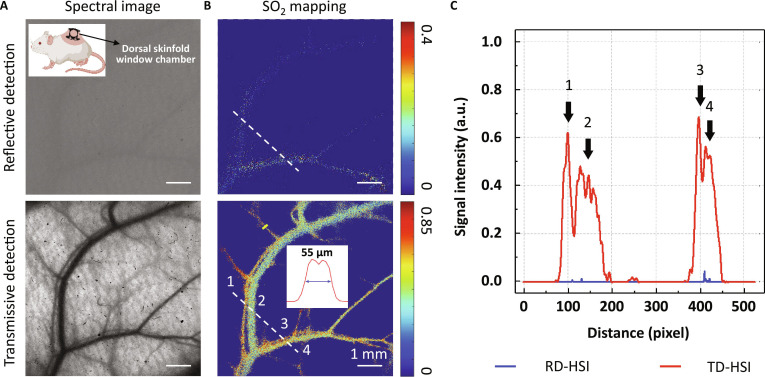
Comparison of TD-HSI and RD-HSI for blood oxygen imaging with in vivo animal experiment. (A and B) Representative spectral images@550 nm (A) and SO_2_ mappings (B) of mouse dorsal skin acquired with reflective or transmissive detection (similar results were obtained from 3 mice). The protrusion on the mouse’s back in (A) indicates the dorsal skinfold window chamber established for in vivo imaging. The plot inset in (B) represents the intensity distribution of the yellow line. (C) Signal intensity distribution on the dotted lines in (B).

Next, we demonstrated the capability of TD-HSI on noninvasively high-resolution SO_2_ mapping in vivo in mice. Figure 3A shows spectral images of mouse dorsal skin captured with 550 nm, at which wavelength it should have had high contrast. However, the reflective-detected image showed unsatisfied imaging quality due to the superficial absorption and scattering. In sharp contrast, the transmissive-detected mode could distinguish the vasculature clearly. Further SO_2_ calculation also demonstrated that TD-HSI could achieve blood oxygen mapping at a single-vessel resolution, while RD-HSI can hardly detect dermal SO_2_ signals through the epidermis (Fig. 3B and C).

### In vivo monitoring of dynamic changes in SO_2_ with TD-HSI

SO_2_ dynamics is essential to reflect physiological or pathological condition [25,26]. Here, we used TD-HSI to investigate blood oxygen response to hypoxia at different body parts. The liquid crystal tunable filter placed in front of the scientific complementary metal-oxide-semiconductor (sCMOS) camera divided the spectrum from 500 nm to 720 nm into 23 narrow bands with a 10-nm interval. The sCMOS camera then captured spectral images at each wavelength with an exposure time of 100 ms, and after calculation, the SO_2_ distribution and changes were obtained.

**Fig. 4.**
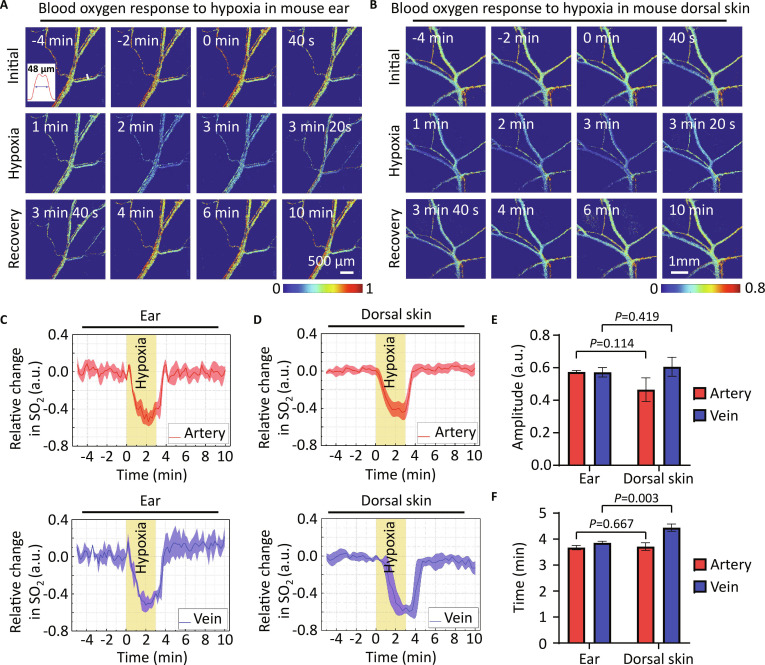
Monitoring of blood oxygen response to hypoxia in mouse ear and dorsal skin with TD-HSI. (A and B) Representative images of blood oxygen mapping in mouse ear (A) and dorsal skin (B) before and after hypoxia stimulus. The plot inset in (A) represents the intensity distribution of the white line. (C and D) Quantification of relative changes in arteriovenous SO_2_ in mouse ear (C) and dorsal skin (D). The yellow shade area indicates the hypoxic stimulus phase. Time = 0 min in the horizontal axis indicates the onset of hypoxia. (E and F) Quantitative comparison of response amplitude (E) and recovery time (F) of SO_2_ in mouse ear and dorsal skin in response to hypoxic stimulus. All values are presented as the means ± standard deviation (*n* = 3 mice for each group); statistical significance was assessed using the independent-sample *t* test.

As shown in Fig. 4A and B, TD-HSI was able to record the changes of SO_2_ in the mouse’s ear and dorsal skin, enabling the observation and measurement of SO_2_ in blood vessels with a diameter of 48 μm. The results revealed that SO_2_ in both arteries and veins decreased during hypoxia and eventually recovered to the normal condition after hypoxia (Fig. 4C and D). Further quantitative analysis showed that the arterial response peak values of ear and dorsal skin were 57.4 ± 0.9% and 48.4 ± 7.7% (*P* = 0.114), while the veinous response peak values were 57.2 ± 2.9% and 60.6 ± 5.8% (*P* = 0.419), respectively (Fig. 4E and F), showing no significant difference in response peak value between mouse ear and dorsal skin. Besides, the arterial blood oxygen levels in mouse ear and dorsal skin returned to the initial levels at 3.67 ± 0.09 min and 3.73 ± 0.18 min, respectively, without significant difference (*P* = 0.667). However, the recovery of venous blood oxygen level in mouse ear was significantly faster than that of dorsal skin (3.86 ± 0.06 min versus 4.44 ± 0.14 min, *P* = 0.003), which might be because it was necessary to give priority to restoring the oxygen supply of the ears with faster metabolism.

### In vivo monitoring of SO_2_ changes during the growth of tumor

**Fig. 5.**
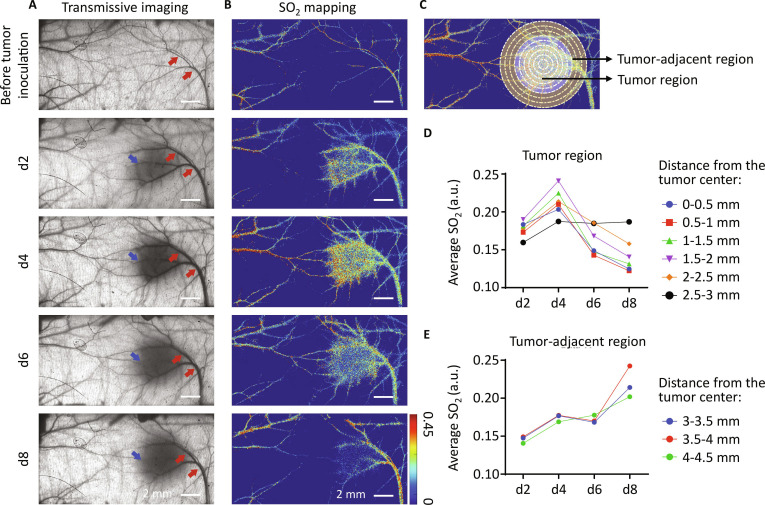
Continuous tracking of mouse dorsal skin before and after tumor inoculation. (A) Spectral images@550 nm of mouse dorsal skin with transmissive detection mode. The blue arrows indicate the location of tumor, and the red arrows indicate the dilated blood vessels during the growth of tumor. (B) SO_2_ mapping in mouse dorsal skin acquired with TD-HSI. (C) Diagram for the calculation of blood oxygen levels at different distances from the tumor center. (D and E) Quantitative analysis of blood oxygen levels in tumor (D) and tumor-adjacent (E) areas. The results were obtained from one mouse.

TD-HSI was then used to monitor in vivo the growth of 4T1 tumor and the corresponding changes of blood oxygen level (Fig. 5). Wide-field imaging at 550 nm revealed that, within 8 d post-tumor implantation, the tumor region steadily grew, accompanied by dilation of adjacent blood vessels (denoted by red arrows in Fig. 5A). This expansion signifies an increased supply of oxygen and nutrients to sustain the rapid tumor growth and establishes potential routes for tumor cell metastasis [27].

As shown in Fig. 5B, TD-HSI enabled to monitor the changes of blood oxygen level during tumor growth. We quantitatively analyzed SO_2_ dynamics at varying distances from the tumor center (refer to Fig. 5C). Our findings indicate that the blood oxygen level in the tumor region progressively increased following tumor implantation, reaching a peak on day 4. However, after 6 d of tumor growth, the blood oxygen level began to decline. Notably, the closer the proximity to the tumor center, the lower the SO_2_ value observed. This downward trend persisted on day 8. Interestingly, in the area adjacent to the tumor, the blood oxygen level steadily rose from day 2 to day 8 of tumor growth (Fig. 5D and E). This increase could be attributed to the enhanced blood oxygen levels in preexisting vessels, ensuring a steady supply of oxygen to the oxygen-deprived tumor.

## Discussion

We have conducted a comprehensive evaluation of the capability of TD-HSI strategy for deep-tissue blood oxygen imaging. Supported by theoretical simulations and experimental verifications, our results demonstrated that TD-HSI offered substantial improvements over conventional RD-HSI in noninvasively acquiring blood oxygen information in deep tissue.

The Monte Carlo algorithm, with its powerful simulation capability and flexible adaptability, has become an effective tool for simulating light propagation in complex organizations [28]. In 2021, Li et al. [29] clarified through Monte Carlo simulations that transmissive detection outperformed reflective detection in acquiring deep signals. In addition, TD-HSI has been used to distinguish different components in tissues and identify lesion areas through the differences in spectral information [30,31]. However, for obtaining oxygen distribution in tissue by HSI, in addition to detecting spectral information from the tissue, it is also necessary to calculate the interaction between light and tissue. In this work, we integrated hyperspectral analysis method with Monte Carlo simulations to more precisely delineate the benefits of TD-HSI, and confirmed in theory that TD-HSI not only extended the depth of SO_2_ measurement but also enhanced the accuracy and sensitivity of detecting changes in SO_2_ within deep tissues.

TD-HSI was applied to map resting-state blood oxygen distribution in mice skin, achieving single-vessel resolution, which demonstrated that transmissive detection improved HSI to extract deep blood oxygen information compared to reflective detection. In addition, TD-HSI was applied to dynamically monitor changes in SO_2_ induced by hypoxia across the ears and dorsal skin of mice, achieving high temporal-spatial resolution. The separate analysis of arterial and venous responses at these sites revealed a notably slower recovery of venous SO_2_ in the dorsal skin post-hypoxia compared to the ear veins, which may be attributed to the higher oxygen consumption in mouse ear than in dorsal skin; therefore, the body preferentially restores the blood oxygen level in the ear to meet its high oxygen demand after the hypoxic stimulation. Assessing the hypoxia tolerance of different tissues and blood vessels and their recovery capabilities can help optimize hypoxia-related medical interventions. Additionally, employing TD-HSI to track the blood oxygen response of peripheral vessels to stimuli under pathological conditions would provide critical insights into disease-induced vascular dysfunction.

Furthermore, TD-HSI was employed to measure in vivo changes in blood oxygen levels during tumor growth. We observed an initial increase in SO_2_ within the tumor region, followed by a subsequent decline. The early rise can primarily be attributed to the angiogenesis, which meets the escalating energy and nutrient demands of the tumor cells. As the tumor progresses, the increased metabolic demands of the tumor cells consume substantial amounts of oxygen. Concurrently, the structural and functional anomalies of microcirculation within the tumor impede effective oxygen delivery, resulting in a hypoxic state of the tumor at the later stage [32]. Sorg et al. [33] also monitored the evolution of tumor hypoxia. Serial fluorescence imaging of 4T1 tumor grown in a dorsal skinfold window chamber was performed to identify all tumor cells and hypoxic fraction. They found that hypoxic tumor cells began to appear by day 4 post-implantation, and almost the entire tumor area became hypoxic by day 8, which basically corroborated our observations and demonstrated the effectiveness of noninvasive and label-free TD-HSI for monitoring changes in tumor blood oxygen levels in vivo.

Admittedly, unlike photoacoustic imaging or OCT, the limited optical penetration depth of TR-HSI restricts its application to thin anatomical regions, such as the hands or ears. Photoacoustic imaging combines the advantages of high optical imaging resolution and deep acoustic penetration, enabling the detection of SO_2_ in deep tissues [34]. By using near-infrared signals, OCT enables high-resolution acquisition of SO_2_ with the depth of several millimeters [35,36]. In future work, adopting near-infrared wavelength illumination or combining in vivo optical clearing methods [37–39] to reduce tissue scattering is expected to further improve the imaging depth and resolution of TD-HSI. In addition, the setup of the HSI system and the hyperspectral analysis method warrant further optimization to improve the SO_2_ measurement accuracy of TD-HSI. For instance, incorporating an angular filter array into the imaging system can refine spectral measurements by selectively accepting ballistic and quasiballistic photons from collimated incident light. Moreover, replacing the multiple linear regression algorithm with a lookup-table-based hyperspectral analysis that considers the multi-layer structure and optical properties of biological tissues might yield higher fitting accuracy [40]. Furthermore, the development of motion artefact removal algorithms to avoid artifacts is also crucial for future expansion of its applications.

In conclusion, TD-HSI stands out relying on a simple system architecture and low costs in conducting noninvasive, large-field, and label-free blood oxygen monitoring with high temporal-spatial resolution. It also presents great potential for miniaturization, promising substantial advances in microcirculation research.

## Materials and Methods

### Monte Carlo simulation and theoretical calculation

Monte Carlo simulation and theoretical calculation were performed to compare the ability of RD-HSI and TD-HSI in detecting blood oxygen signal theoretically. Firstly, tissue models with and without vascular layer were constructed. The geometric and optical parameters for 2 tissue models were presented in Tables S1 and S2, respectively [41,42]. As the HSI measurement of SO_2_ was primarily based on the absorption of different wavelengths by the blood, tissue models were simplified where parameters other than the absorption coefficient of the vascular layer did not vary with wavelength. The wavelength-dependent absorption coefficient of the blood vessel $\mu_{a\_\mathrm{BV}}^{\lambda }$ was calculated using Eq. (1) [40]:

$$\mu_{a\_\mathrm{BV}}^{\lambda }=B\cdot \left(\varepsilon_{\mathrm{oxy}}^{\lambda }\cdot {\mathrm{SO}}_{2\_\text{preset}}+\varepsilon_{\text{deoxy}}^{\lambda }\cdot \left(1-{\mathrm{SO}}_{2\_\text{preset}}\right)\right)$$ (1)

where B is the hemoglobin concentration of the vascular layer. $\varepsilon_{\mathrm{oxy}}^{\lambda }$ and $\varepsilon_{\text{deoxy}}^{\lambda }$ are wavelength-dependent molar extinction coefficients of oxygenated hemoglobin and deoxygenated hemoglobin, respectively. Wavelength λ = 500, 510, …, 720 nm. SO_2_preset_ is the pre-set SO_2_ value. To simulate arteries and veins, SO_2_preset_ is set to be 98% and 75%, respectively [43].

As illustrated in Fig. S2, the Monte Carlo technique was performed to simulate the propagation of a flat beam with a certain wavelength $I_{0}^{\lambda }$ (diameter: 4 cm, light intensity: 0.1 J, λ = 500, 510, …, 720 nm) in the tissue model with the vascular layer. The radial distribution of the reflected light $I_{R}^{\lambda }$ or transmitted light $I_{T}^{\lambda }$ was then calculated. Similarly, the radial distribution of the reflected light $I_{R\_\text{Background}}^{\lambda }$ or transmitted light $I_{T\_\text{Background}}^{\lambda }$ from a flat beam incident on tissue without the vascular layer was also acquired.

Next, a mathematical calculation was performed to extract the SO_2_ value. In the reflective detection, the absorbance of the vascular layer was calculated according to Eq. (2) [18]:

$$\begin{array}{c} A_{\text{vessel}}^{\lambda }=A^{\lambda }-A_{\text{Background}}^{\lambda }=\text{log}\frac{1}{R^{\lambda }}-\text{log}\frac{1}{R_{\text{Background}}^{\lambda }}\\ \;=\text{log}\frac{1}{R^{\lambda }/R_{\text{Background}}^{\lambda }} \end{array}$$ (2)

where $A^{\lambda }$, $A_{\text{Background}}^{\lambda }$, and $A_{\text{vessel}}^{\lambda }$ are the absorbance of tissue with vascular layer, tissue without vascular layer, and vascular layer, respectively. $R^{\lambda }$ and $R_{\text{Background}}^{\lambda }$ represent the reflectance at tissues containing and without vascular layers, respectively.

In the simulation, $R^{\lambda }/R_{\text{Background}}^{\lambda }$ was equivalent to $I_{R}^{\lambda }/I_{R\_\text{Background}}^{\lambda }$, according to Eq. (3):

$$\frac{R^{\lambda }}{R_{\text{Background}}^{\lambda }}=\frac{I_{R}^{\lambda }/I_{0}^{\lambda }}{I_{R\_\text{Background}}^{\lambda }/I_{0}^{\lambda }}=\frac{I_{R}^{\lambda }}{I_{R\_\text{Background}}^{\lambda }}$$ (3)

The parameter $A_{\text{vessel}}^{\lambda }$ could also be expressed as Eq. (4) according to the modified Beer–Lambert law:

$$A_{\text{vessel}}^{\lambda }=B\cdot \left(\varepsilon_{\mathrm{oxy}}^{\lambda }\cdot {\mathrm{SO}}_{2}\cdot L+\varepsilon_{\text{deoxy}}^{\lambda }\cdot \left(1-{\mathrm{SO}}_{2}\right)\cdot L\right)+\xi$$ (4)

where L is the optical path and ξ represents the influence of other substances other than hemoglobin on absorbance.

$I_{R}^{\lambda }$ and $I_{R\_\text{Background}}^{\lambda }$ in Eqs. (3) and (4) were replaced by $I_{T}^{\lambda }\;\text{and}$ $I_{T\_\text{Background}}^{\lambda }$, respectively, in the simulation of transmissive detection.

The modified Beer–Lambert law equations were expressed in matrix form, as shown in Eq. (5):

$$\left[\begin{array}{c} A\left(\lambda_{1}\right)\\ A\left(\lambda_{2}\right)\\ \ldots \\ A\left(\lambda_{23}\right) \end{array}\right]\approx \left[\begin{array}{ccc} 1 & \varepsilon_{\mathrm{oxy}}^{\lambda_{1}} & \varepsilon_{\text{deoxy}}^{\lambda_{1}}\\ 1 & \varepsilon_{\mathrm{oxy}}^{\lambda_{2}} & \varepsilon_{\text{deoxy}}^{\lambda_{2}}\\ \ldots & \ldots & \ldots \\ 1 & \varepsilon_{\mathrm{oxy}}^{23} & \varepsilon_{\text{deoxy}}^{23} \end{array}\right]\left[\begin{array}{c} \xi \\ B\cdot {\mathrm{SO}}_{2}\cdot L\\ B\cdot \left(1-{\mathrm{SO}}_{2}\right)\cdot L \end{array}\right]$$ (5)

making

$$\begin{array}{c} A=\left[\begin{array}{c} A\left(\lambda_{1}\right)\\ A\left(\lambda_{2}\right)\\ \ldots \\ A\left(\lambda_{23}\right) \end{array}\right],X=\left[\begin{array}{ccc} 1 & \varepsilon_{\mathrm{oxy}}^{\lambda_{1}} & \varepsilon_{\text{deoxy}}^{\lambda_{1}}\\ 1 & \varepsilon_{\mathrm{oxy}}^{\lambda_{2}} & \varepsilon_{\text{deoxy}}^{\lambda_{2}}\\ \ldots & \ldots & \ldots \\ 1 & \varepsilon_{\mathrm{oxy}}^{23} & \varepsilon_{\text{deoxy}}^{23} \end{array}\right],C=\left[\begin{array}{c} \xi \\ B\cdot {\mathrm{SO}}_{2}\cdot L\\ B\cdot \left(1-{\mathrm{SO}}_{2}\right)\cdot L \end{array}\right] \end{array}$$

The matrix shown in Eq. (5) could be simplified to Eq. (6):

A≈X·C (6)

The non-negative least square method was employed to solve the matrix C in Eq. (5), and SO_2_ could be calculated according to Eq. (7):

$${\mathrm{SO}}_{2}=C\left(2\right)/\left(C\left(2\right)+C\left(3\right)\right)$$ (7)

Whether to accept the fitting value as the SO_2_ value of each pixel was judged according to the Pearson correlation coefficient. The fitting value was accepted when the Pearson correlation coefficient was greater than or equal to 0.9; otherwise, the value was rejected [40].

### TD-HSI and conventional RD-HSI

HSI systems based on reflective or transmissive detection were constructed for blood oxygen imaging, respectively (Fig. 2A). For TD-HSI, a light-emitting diode (LED) light (GCI-060411, Daheng Optics, China) was positioned beneath the sample, with the emitted light beam collimated by a lens. The light then passed through the entire sample and was collected with a stereomicroscope (SZX12, Sunny, China). Conversely, in the conventional RD-HSI system, the LED was obliquely positioned above the sample and the reflected light entered the stereomicroscope from the sample surface. For SO_2_ mapping, the illumination power on the sample was set to 9 mW (5 mW/cm^2^) for both TD-HSI and RD-HSI. A computer-controlled liquid crystal tunable filter (CRi Varispec VIS, Perkin Elmer, USA) was utilized to divide the continuous spectrum ranging from 500 to 720 nm into 23 narrow bands (7-nm bandwidth) with a 10-nm wavelength interval. Corresponding spectral images were acquired by sCMOS (pco.panda 4.2, PCO, Germany), and the exposure time for each wavelength was 100 ms. Reference images were also obtained. For the TD-HSI, the sample was removed and the camera imaged the light source directly. For the conventional RD-HSI, a diffuse reflectance standard (SRS-99-020, Labsphere, USA) was placed at the location of the sample and imaged by the detector. The dark noise was measured with the light source and the detector shutter closed. The wavelength bands in the acquisition of reference images and dark images were consistent with those used in the imaging of the sample. The exposure time was adjusted to ensure that the detector received the same light intensity when comparing the imaging quality of 2 detection modes.

The SO_2_ mapping of the sample could be derived by data processing of the raw hyperspectral dataset, reference images, and dark images using the mathematical calculation described in Eqs. (2) to (7), in which Eq. (3) should be modified as follows for real imaging.

Rλ/RBackgroundλ=Iλ−InoiseλIreferenceλ−InoiseλIBackgroundλ−InoiseλIreferenceλ−Inoiseλ (8)

where $I^{\lambda }$, $I_{\text{reference}}^{\lambda }$, and $I_{\text{noise}}^{\lambda }$ represent pixel intensities of spectral image, reference image, and noise image taken by RD-HSI or TD-HSI at wavelength λ, respectively.

### Construction of tissue phantom

To construct the tissue phantom, 1% intralipid (Fresenius Kabi, China) in the culture dish was used to simulate the turbid tissue. The capillary tube (inner diameter: 0.5 mm, outer diameter: 1.0 mm) was filled with fresh blood taken from mice and mixed with heparin sodium, which was used to simulate the blood vessel. The capillary glass tube was inserted diagonally into the culture dish, and the thickness of intralipid was fixed at 2 mm to simulate the tissue whose thickness was constant, while the depth of the blood vessel inside varied continuously.

### Animal treatment

Mice (Balb/c, female) were anesthetized with 1.5% isoflurane and placed on the heating pad to maintain a constant body temperature. The hair on mouse ears and dorsal skin was shaved and removed completely using the hair removal cream. For imaging, the edges of mouse ears were affixed to slides with glue, while the dorsal skin was lifted into a double layer and fixed with an aluminum bracket (the dorsal skinfold window chamber, which is commonly used in intravital subcutaneous microscopy [44,45]). Then, the mice were placed on the imaging platform and the blood oxygen mappings in mouse ears or dorsal skin were acquired with RD-HSI or TD-HSI. All animal procedures were approved by the Experimental Animal Management Ordinance of Hubei Province, China (no. 1000639903375), and carried out following the guidelines for humane care of animals.

To assess the ability of TD-HSI in monitoring dynamic changes in SO_2_, we monitored the blood oxygen response to hypoxia stimulus in mouse ears and dorsal skin, respectively. Firstly, the mice were anesthetized by inhalation of a mixture of 1.5% isoflurane, 20% oxygen, and 78.5% nitrogen (0.6 l min^−1^). When the mice stayed in a stable state, TD-HSI was performed continuously. Five minutes later, the gas inhaled by mice was converted to a mixture of 1.5% isoflurane, 9.85% oxygen, and 88.65% nitrogen to induce hypoxia. After 3 min, the mice inhaled the mixture of 1.5% isoflurane, 20% oxygen, and 78.5% nitrogen again to recover. Blood oxygen mappings of mouse ears and dorsal skin at every time point were acquired using MATLAB, and the relative changes in SO_2_ ($∆{\mathrm{SO}}_{2}$) were calculated using Eq. (9).

$$\Delta {\mathrm{SO}}_{2}=\left(I_{i}-I_{\mathrm{BL}}\right)/I_{\mathrm{BL}}$$ (9)

where $I_{i}$ is the SO_2_ value at a certain time point and $I_{\mathrm{BL}}$ is the baseline value, that is, the average SO_2_ value before hypoxia stimulus.

The maximal decrease in SO_2_ during hypoxia was called the peak response. Timing from the beginning of hypoxia, the time it takes for SO_2_ to return to the initial value was termed the response time.

### TD-HSI for transplanted tumor model

To establish the transplanted tumor model, the mouse (Balb/c, female, 6 weeks old) was anesthetized. Then, the dorsal skin was shaved and cleaned with alcohol and normal saline. 4T1 mouse mammary carcinoma cells in the logarithmic growth phase were prepared into phosphate-buffered saline suspension and subcutaneously inoculated into the back of mouse (150 μl, at a density of 6 × 10^3^ cells μl^−1^). The day of tumor inoculation was defined as day 0.

TD-HSI system was applied to image the transplanted tumor and its surrounding area before and at days 2, 4, 6, and 8 after tumor inoculation, respectively, with the same imaging parameters. The exposure time was 100 ms, and the magnification was 0.75×.

To calculate the changes of blood oxygen levels in tumor and surrounding areas during tumor growth, concentric circles were drawn in the blood oxygen distribution images at 0.5-mm intervals within 4.5 mm from the tumor center. The average ${\mathrm{SO}}_{2}$ value in each annulus was calculated. Since the tumor grew within a circular area with a radius of 3 mm during the monitoring period, this range was defined as the tumor area, and the area 3 to 4.5 mm away from the tumor center was defined as the tumor-adjacent area.

## Data Availability

The data that support the findings of this study are available from the corresponding authors upon reasonable request.
