# Supplementary material for: Transmissive-Detected Hyperspectral Imaging for Single-Vessel-Resolution Blood Oxygen Mapping
Source: BME Front. 2025 Dec 16;6:0211. doi: 10.34133/bmef.0211 (PMC12707973; doi:10.34133/bmef.0211)
Supplement: Supplementary 1 — Figs. S1 and S2 Tables S1 and S2 [file bmef.0211.f1.docx]

**Transmissive-detected Hyperspectral Imaging for Single-vessel-resolution Blood Oxygen Mapping**

**Shaojun Liu^1, 2#^,** **Qing Xia^1#^, Yuwei Du^1^, Tingting Yu^1^, Dongyu Li^1, 3*^, and Dan Zhu^1, 2*^**

^1^MoE Key Laboratory for Biomedical Photonics, Wuhan National Laboratory for Optoelectronics-Advanced Biomedical Imaging Facility, Huazhong University of Science and Technology, 430074 Wuhan, Hubei, China.

^2^Optics Valley Laboratory, 430074 Wuhan, Hubei, China.

^3^School of Optical Electronic Information, Huazhong University of Science and Technology, 430074 Wuhan, Hubei, China.

**^*^**Address correspondence to: li[_dongyu@hust.edu.cn](mailto:_dongyu@hust.edu.cn) and [dawnzh@mail.hust.edu.cn](mailto:dawnzh@mail.hust.edu.cn)

**^#^**These authors contributed equally to this work.

Table S1. Parameters for the tissue model with vascular layer

| **Skin layer** | $\text{n}$ | $\text{μ}_{\text{a}} \text{(}\text{cm}^{\text{-1}}\text{)}$ | $\text{μ}_{\text{s }}\text{(}\text{cm}^{\text{-1}}\text{)}$ | $\text{g}$ | **Thickness**  **(**$\text{cm}$**)** | **Radius**  **(**$\text{cm}$**)** |
| --- | --- | --- | --- | --- | --- | --- |
| Dermis | 1.40 | 0.28 | 80.6 | 0.82 | $\text{d}_{\text{upper dermis}}$ | 1 |
| Blood vessel | 1.39 | $\text{μ}_{\text{a\_BV}}^{\text{λ}}$ | 85.3 | 0.83 | 0.01 | 1 |
| Dermis | 1.40 | 0.28 | 80.6 | 0.82 | $\text{d}_{\text{lower dermis}}$^a^ | 1 |

^a^The tissue model has a total thickness of 2 mm. The thickness of the tissue above the vascular layer, denoted as $\text{d}_{\text{upper dermis}}$, was varied as 0 mm, 0.5 mm, 1 mm, and 1.5 mm, respectively. Correspondingly, the thickness of the tissue below the vascular layer, denoted as $\text{d}_{\text{lower dermis}}$, was set to be 1.9 mm, 1.4 mm, 0.9 mm, and 0.4 mm, respectively.

Table S2. Parameters for the tissue model without vascular layer

| **Skin layer** | $\text{n}$ | $\text{μ}_{\text{a}} \text{(}\text{cm}^{\text{-1}}\text{)}$ | $\text{μ}_{\text{s }}\text{(}\text{cm}^{\text{-1}}\text{)}$ | $\text{g}$ | **Thickness**  **(**$\text{cm}$**)** | **Radius**  **(**$\text{cm}$**)** |
| --- | --- | --- | --- | --- | --- | --- |
| Dermis | 1.40 | 0.28 | 80.6 | 0.82 | 0.2 | 1 |


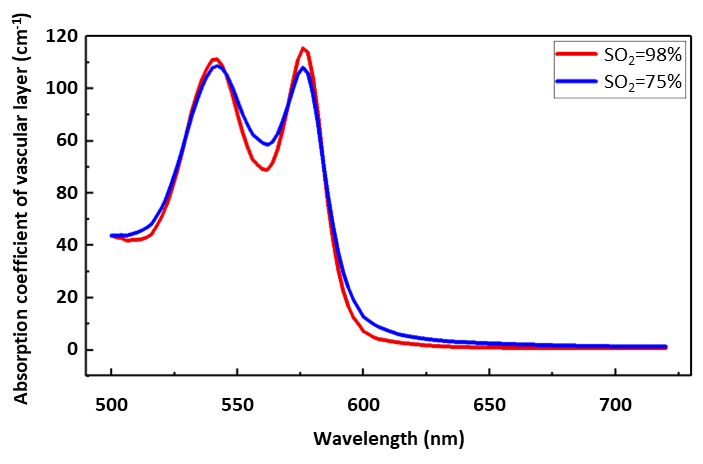


**Fig. S1.** Theoretical absorption spectra of arterial (SO_2_=98%) and venous (SO_2_=75%) blood.


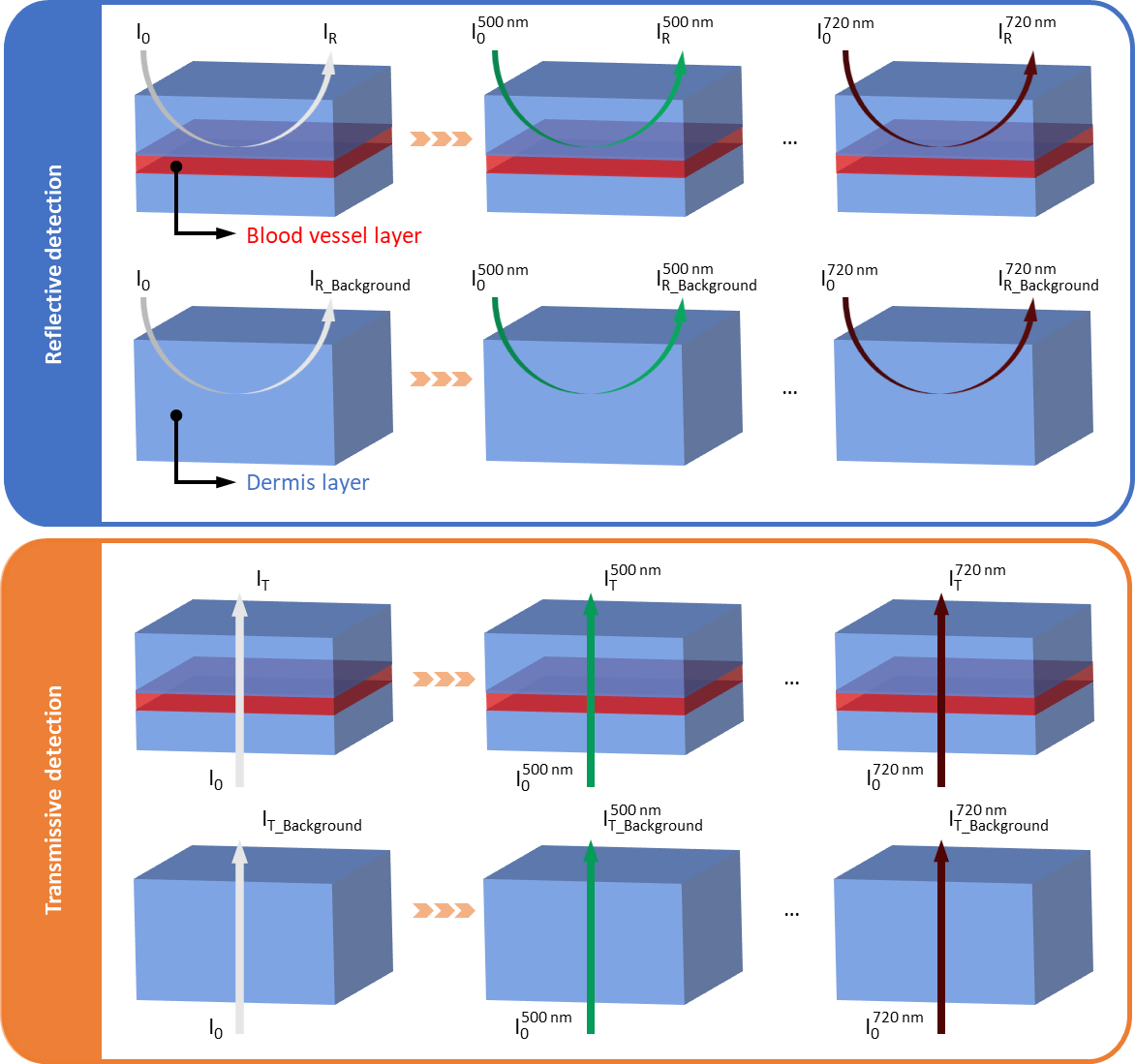


**Fig. S2.** Schematic diagram of Monte Carlo technique for the simulation of HSI with reflective and transmissive detection modes.
